# A new co-ultramicronized composite including palmitoylethanolamide and luteolin to prevent neuroinflammation in spinal cord injury

**DOI:** 10.1186/1742-2094-10-91

**Published:** 2013-07-23

**Authors:** Irene Paterniti, Daniela Impellizzeri, Rosanna Di Paola, Michele Navarra, Salvatore Cuzzocrea, Emanuela Esposito

**Affiliations:** 1 Department of Biological and Environmental Sciences, University of Messina, Viale Ferdinando Stagno D’Alcontres, 98166 Messina, Italy; 2 Pharmaco-Biological Department, University of Messina, Viale Annunziata, 98100 Messina, Italy

**Keywords:** Palmitoylethanolamide, Luteolin, Antioxidant action, Neuroinflammation, Spinal cord injury

## Abstract

**Background:**

It has recently been demonstrated that palmitoylethanolamide (PEA), an endogenous lipid amide belonging to the *N*-acylethanolamine family, exerts neuroprotection in central nervous system (CNS) pathologies. In recent studies, we have demonstrated that treatment with PEA significantly reduced inflammatory secondary events associated with spinal cord injury (SCI). Since oxidative stress is considered to play an important role in neuroinflammatory disorders, in the present work we studied a new composite, a formulation including PEA and the antioxidant compound luteolin (Lut), subjected to an ultramicronization process, co-ultraPEALut. We investigated the effect of co-ultraPEALut (in the respective fixed doses of 10:1 in mass) in both an *ex vivo* organotypic spinal cord culture model and an *in vivo* model of SCI.

**Methods:**

For the organotypic cultures, spinal cords were prepared from mice at postnatal day 6 and were cut into transverse slices of 400 μm thickness to generate the lumbar organotypic slice cultures. After 7 days of culturing, the slices were mechanically injured onto the center of the slice and the co-ultraPEALut was applied at different concentrations (0.00009, 0.0009 and 0.009 g/l) 1 hour before damage. For *in vivo* studies, SCI was induced in mice through spinal cord compression by the application of vascular clips (force of 24 g) to the dura via a four-level T5 to T8 laminectomy, and co-ultraPEALut (1 mg/kg ip) was administered at 1 and 6 hours after SCI. At 24 hours after SCI, mice were sacrificed and the spinal cords were collected for further evaluation. Additional animals were treated similarly and sacrificed 10 days after SCI.

**Results:**

Pretreatment with co-ultraPEALut significantly reduced cyclooxygenase-2 (COX-2) and inducible nitric oxide synthase (iNOS) expression in a concentration-dependent manner, restored neuronal nitric oxide synthase (nNOS) expression at all three tested concentrations, and protected cells by cell death (MTT assay) in spinal cord organotypic cultures. Moreover, we demonstrated *in vivo* that co-ultraPEALut 1 mg/kg reduced the severity of trauma induced by compression and improved the motor activity evaluated at 10 days post-injury.

**Conclusion:**

The present study demonstrates that the protective effect of PEA on SCI-associated neuroinflammation could be improved by co-ultramicronization with Lut possibly due to its antioxidant properties.

## Background

Interest in neuroinflammation has grown rapidly over the past decade, driven by increasing evidence for its role in the development of several important neurodegenerative diseases, such as Alzheimer’s disease, Parkinson’s disease, stroke, traumatic brain injury, spinal cord injury (SCI) and demyelinating disorders, as well as pathologies associated with central nervous system (CNS) infections. Traumatic injuries to the spinal cord frequently cause permanent neurological disabilities and yet there is no effective therapeutic option to improve functional recovery [1-3]. SCI-induced inflammation may result in further reduction in functional recovery of the development of scar tissue, as well as necrosis or apoptosis of neurons and oligodendrocytes, which occurs for at least 2 weeks post-injury, and are rapidly lost at the injury site [4-6]. In the last decade, strategies that non-selectively suppress inflammation have had varying effects on outcomes after experimental SCI. This variability might, at least in part, be attributed to the unique roles of inflammatory cells in the processes of injury and recovery. The latter motivates research efforts to identify the mechanisms underlying inflammation during SCI and to test new compounds to control them.

In this study, using an *ex vivo* model of spinal cord organotypic cultures and an *in vivo* compression model of SCI in mice, we analyzed the neuroprotective properties of a co-ultramicronized combination product based on association of palmitoylethanolamide (PEA), an endogenous fatty acid amide belonging to the *N*-acylethanolamine family, with the flavonoid luteolin (Lut). PEA has been shown to inhibit peripheral inflammation and mast cell degranulation [7], as well as exerts antinociceptive effects in rats and mice [8,9]. These actions are in part mediated by the activation of peroxisome proliferator-activated receptors (PPARs), accompanied by a decrease in neutrophil influx and a decrease in expression of pro-inflammatory proteins, such as inducible nitric oxide synthase (iNOS) and cyclooxygenase-2 (COX-2) [10,11]. Moreover, our previous works clearly demonstrated that treatment with PEA at 10 mg/kg significantly reduced the inflammation process associated with experimental SCI in mice [12] and in a traumatic brain injury (TBI) model [13]. However, PEA lacks a direct antioxidant capacity to prevent the formation of free radicals, and to counteract the damage of DNA, lipids and proteins, all of which are important events occurring in diseases of the CNS, such as SCI.

Lut, a common flavonoid present in many plants, has strong antioxidant and pharmacological activities, including a memory-improving effect. It displays excellent radical scavenging and cytoprotective properties, particularly when tested in complex biological systems where it can interact with other antioxidants, such as vitamins. Lut displays specific anti-inflammatory effects, which are only partly explained by its antioxidant capacities. The anti-inflammatory activity of Lut includes activation of antioxidative enzymes, suppression of the nuclear factor (NF)-κB pathway and inhibition of pro-inflammatory substances. *In vivo*, Lut reduces increased vascular permeability and is effective in animal models of CNS inflammation [14,15].

Thus, in this study we assessed the neuroprotective effect of a co-ultramicronized PEA with Lut association (co-ultraPEALut) and verified if the new composite exerts more potent effects compared to the single compounds.

## Methods

### Co-ultramicronization process of palmitoylethanolamide (PEA) and luteolin (Lut)

**Figure 1:**
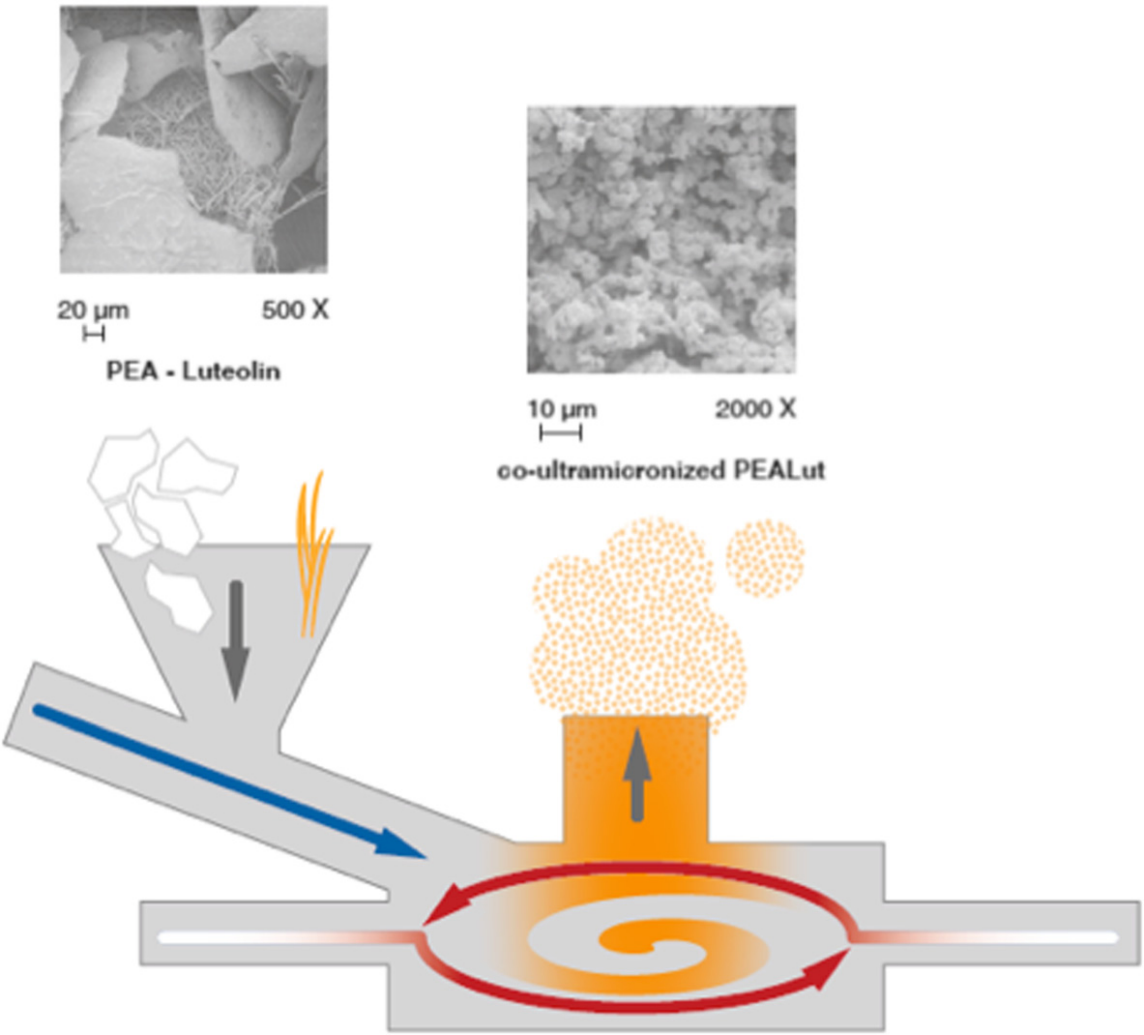
**Schematic representation of the co**-**ultramicronization process.** Once coarse particles of PEA and Lut enter the system through the feed funnel (downwards gray arrow), they are forced to the grinding chamber by compressed air (blue arrow). There, high-speed rotation sustained by grid air (white to red arrows) subjects the coarse mixture to particle-on-particle impact reduction. Centrifugal force holds larger particles in the grinding area, while centripetal force drives preselected-sized fines toward the center for discharge. Depending on its size, the co-ultramicronized particle remains inside the grinding chamber until it has a size that allows the exiting process gas to transport it out, depending on the vortex finder (upwards grey arrow). The scanning electron microscopy views represent the naïve mixture of the two compounds (left-hand side) and the resulting co-ultramicronized composite, ‘co-ultraPEALut’ (right-hand side), respectively. Lut, luteolin; PEA, palmitoylethanolamide.

The co-ultramicronization process was performed in jet mill equipment (Sturtevant Inc., 348 Circuit Street Hanover, MA, USA), endowed with a chamber of 300 mm in diameter, operated with ‘spiral technology’ and driven by compressed air at 10 to 12 bars (Figure 1). The crashing was determined by the high number of collisions that occurred among particles as a result of the high level of kinetic (not mechanical) energy. This process is effective not only in reducing the product particle size, but also in modifying the crystalline structure. Observations by scanning electron microscopy showed an intimate intermixing of the two components of the composite, while analysis of differential scanning calorimeter (DSC) and X-ray diffraction (XRD) documented the transformation in a new crystalline form different from the original two, definable with ‘a higher energy content form’. The composite showed the following particle size distribution: 96% <10 μm; 80% <5 μm; and 40% <2 μm. Co-ultraPEALut was dissolved in Pluronic F68 (Sigma-Aldrich, St Louis, MO, USA) and used at a concentrations of:

1) 0.00009 g/l, containing PEA and Lut at 0.27 and 0.027 μM, respectively, ‘co-ultraPEALut (0.27 + 0.027 μM)’.

2) 0.0009 g/l, containing PEA and Lut at 2.7 and 0.27 μM, respectively, ‘co-ultraPEALut (2.7 + 0.27 μM)’.

3) 0.009 g/l, containing PEA and Lut at 27 and 2.7 μM, respectively, ‘co-ultraPEALut (27 + 2.7 μM)’.

The stock solutions of 9 and 0.9 g/l were prepared in 10% Pluronic F68, and required sonication. The final concentration of Pluronic F68 was <0.05%.

### Animals

CD1 mice (Harlan, Milan, Italy) were housed in a controlled environment, and provided with standard rodent chow and water. Animal care was in compliance with Italian regulations on protection of animals used for experimental and other scientific purposes (DM 116192), as well as with the European Economic Community (EEC) regulations (OJ of EC L 358/1 12/18/1986). All experimental research on animals followed the internationally recognized guidelines.

### Preparation of spinal cord organotypic slice cultures

Spinal cord slice cultures were prepared from mouse spinal cord at postnatal day 6, as previously described [16]. In brief, after decapitation with large bandage scissors, the dorsal skin and musculature of the trunk were removed along the midline, using small scissors and surgical forceps. Subsequently, a longitudinal laminectomy was performed from the cervical to the lumbar region of the vertebral column, the dura mater was incised, and the spinal cord was dissected from the denticulate ligaments and immediately placed in ice-cold dissecting media (pH 7.15). The remnants of the surrounding dura mater were removed under microscopic control. Next, the spinal cord was cut into transverse slices of 400 μm thickness using a tissue chopper (McIlwain, ON, Canada) to generate the spinal cord organotypic slice cultures and placed into a sterile petri dish with Earle’s balanced salt solution. Only sections from the thoracic spinal cord were used in the preparation. Cervical and lumbar sections were excluded from the cultures on the basis of their non-uniform cross-sectional size. To obtain reliable data with analysis of cell death, we cultured only thoracic slices that were very consistent in cross-sectional size and in this way each animal generated up to eight usable slices. Finally, spinal slices were transferred onto Millicell-CM cultured plate inserts (Millipore, Billerica, MA, USA). The inserts were placed into wells of a 6-well plate containing 1.5 ml of antibiotic-free medium, containing: 50% MEM with Earle’s balanced salt solution and glutamine; 25% Hank’s balanced salt solution; and 25% horse serum supplemented with 20 mM 4-(2-hydroxyethyl)-1-piperazineethanesulfonic acid (HEPES) sodium salt and 6 mg/ml D-glucose (Gibco, Carlsbad, CA, USA). Slices were incubated at 37°C for 7 days and the medium was changed three times a week. Organotypic cultures were examined on a daily basis to observe general structural integrity (white and gray matter) and neurite outgrowth.

### Treatments

After 7 days of stabilization of growth, cultures were divided into the following groups:

1) Control (Ctr): intact spinal cord slices were cultured with normal culture medium and treated with vehicle only.

2) Damage: spinal cord slices were sagittally cut with a blade under microscopic control [16].

3) Damage + co-ultraPEALut (0.27 + 0.027 μM): spinal cord slices were sagittally cut, as described, and the co-ultramicronized composite PEALut was applied at the concentration of 0.00009 g/l, containing PEA and Lut at 0.27 and 0.027 μM, respectively, and placed in culture medium 1 hour before injury.

4) Damage + co-ultraPEALut (2.7 + 0.27 μM): spinal cord slices were sagittally cut, as described, and the co-ultramicronized composite PEALut was applied at 0.0009 g/l, containing PEA and Lut at 2.7 and 0.27 μM, respectively, and placed in culture medium 1 hour before injury.

5) Damage + co-ultraPEALut (27 + 2.7 μM): spinal cord slices were sagittally cut, as described, and PEALut was applied at 0.009 g/l, containing PEA and Lut at 27 and 2.7 μM, respectively, and placed in culture medium 1 hour before injury.

6) Damage + PEA (1 μM): spinal cord slices were sagittally cut, as described, and PEA alone was applied at 1 μM, and placed in culture medium 1 hour before injury.

7) Damage + Lut (0.1 μM): spinal cord slices were sagittally cut, as described, and Lut alone was applied at 0.1 μM, and placed in culture medium 1 hour before injury.

8) Damage + PEA + Lut association: spinal cord slices were sagittally cut, as described, and PEA and Lut association was applied at 1 and 0.1 μM, respectively, and placed in culture medium 1 hour before injury. The concentration of 1 μM of PEA and 0.1 μM of Lut were chosen to maintain the ratio of 10:1 of the composite co-ultraPEALut.

In all of the groups, the compounds were left in a culture medium for 24 hours after injury. Spinal cord slices were then used for western blot analysis, nitrite production and 3-(4,5- dimethylthiazol-2-yl)-2,5-diphenyltetrazolium bromide (MTT) assay.

### Viability of organotypic cultures by tetrazolium dye

At 24 hours after mechanical damage, viability of organotypic cultures was assessed by using a mitochondria-dependent dye for live cells (tetrazolium dye; MTT) to formazan. Cultures were incubated at 37°C with MTT (0.2 mg/ml) for 1 hour. Culture medium was removed by aspiration and the cells were lysed with dimethyl sulfoxide (DMSO; 100 μl). The extent of reduction of MTT to formazan within cells was quantified by the measurement of optical density at 550 nm (OD_550_) with the microplate reader [17].

### Measurement of nitrite levels

Total nitrite levels, as an indicator of nitric oxide (NO) synthesis, were measured in the supernatant. Briefly, the nitrate in the medium was reduced to nitrite by incubation with nitrate reductase (670 mU/ml) and β-nicotinamide adenine dinucleotide 3-phosphate (160 mM) at room temperature for 3 hours. The total nitrite concentration was then measured using the Griess reaction by adding 100 μl Griess reagent (0.1% (w/v) *N*-(1-Naphthyl) ethylenediamine dihydrochloride in H_2_O and 1% (w/v) sulfanilamide in 5% (v/v) concentrated H_3_PO_4_; volume 1:1) to the 100 μl sample. OD_550_ was measured using an enzyme-linked immunosorbent assay (ELISA) microplate reader (Tecan, Männedorf, Switzerland). Nitrite concentrations were calculated by comparison with OD_550_ of standard solutions of sodium nitrite prepared in H_2_O.

### Western blot analysis

Spinal cord slices were pooled (three slices) and homogenized in buffer containing 50 mM Tris, pH 7.4, 1 mM ethylenediaminetetraacetic acid (EDTA), 1 mM dithiothreitol and 0.2% Triton X-100, containing protease inhibitor cocktail (Roche, Basel, Switzerland). Samples were heated to 95°C for 5 minutes, and equal amounts of protein (50 μg) were separated on 18% SDS-PAGE gel and transferred to nitrocellulose membrane (Whatman, Dassel, Germany). The specific antibodies anti-neuronal nitric oxide synthase (nNOS; 1:1,000; Cell Signaling, Beverly, MA, USA), anti-iNOS (1:1,000; Cell Signaling), anti-COX-2 (1:500; Cayman Chemical, Ann Arbor, MI, USA), anti-PPARα (1:500; Santa Cruz Biotechnology, Dallas, TX, USA), anti-PPARβ (1:500; Santa Cruz Biotechnology) and anti-PPARγ (1:500; Santa Cruz Biotechnology) were solubilized in 1 × PBS, 5% (w/v) non-fat dried milk and 0.1% Tween 20 PBS-Milk-Tween (PMT)) at 4°C overnight. Membranes were incubated with peroxidase-conjugated bovine anti-mouse immunoglobulin G (IgG) secondary antibody or peroxidase-conjugated goat anti-rabbit IgG (1:2,000; Jackson ImmunoResearch, West Grove, PA, USA) for 1 hour at room temperature. To ascertain that blots were loaded with equal amounts of protein lysates, membranes were also incubated in the presence of the antibody against β-actin (1:1,000; Sigma-Aldrich).

Signals were detected with enhanced chemiluminescence (ECL) detection system reagent according to the manufacturer’s instructions (SuperSignal West Pico Chemiluminescent Substrate, Thermo Fisher Scientific, Waltham, MA, USA). The relative expression of the protein bands was quantified by densitometry with Gel Logic 2200 PRO software (Carestream Health, Rochester, NY, USA) and standardized to β-actin levels. Images of blot signals (8 bit/600 dpi resolution) were imported to analysis software (ImageQuant TL, v2003, Amersham Biosciences, Piscataway, NJ, USA). A preparation of commercially available molecular weight markers (Precision Plus Protein Standard, Bio-Rad, Hercules, CA, USA), consisting of proteins of molecular weight 10 to 250 kDa, was used to define molecular weight positions and as reference concentrations for each molecular weight.

### Spinal cord injury (SCI)

Mice were anesthetized with intraperitoneal administration of ketamine and xylazine (2.6 and 0.16 mg/kg body weight, respectively). A longitudinal incision was made on the midline of the back, exposing the paravertebral muscles, as previously described [18]. These muscles were dissected away, the spinal cord was exposed via a four-level T5 to T8 laminectomy and SCI was produced by extradural compression at T6 to T7 level, using an aneurysm clip with a closing force of 24 g. In all injured groups, the spinal cord was compressed for 1 minute. Sham animals were only subjected to laminectomy. Following surgery, 1.0 cm^3^ of saline was administered subcutaneously in order to replace the blood volume lost during the surgery. During recovery from anesthesia, mice were placed on a warm heating pad and covered with a warm towel. The mice were individually housed in a temperature-controlled room at 27°C. Food and water were provided to the mice *ad libitum*. During this time period, the animals’ bladders were manually voided twice a day until the mice were able to regain normal bladder function.

### Experimental groups and treatments

Mice were randomly allocated into the following groups:

1) Sham + vehicle: mice were subjected to laminectomy but the aneurysm clip was not applied and treated intraperitoneally with vehicle (n = 30).

2) SCI + vehicle: mice were subjected to laminectomy and the aneurysm clip was applied (n = 30).

3) SCI + PEA: mice were subjected to SCI and administered PEA at the dose of 1 mg/kg intraperitoneally at 1 and 6 hours after SCI (n = 30).

4) SCI + PEA + Lut association: mice were subjected to SCI and administered PEA 0.9 mg/kg plus Lut 0.1 mg/kg as a single treatment combination to obtain a final dose of 1 mg/kg, administered intraperitoneally at 1 and 6 hours after SCI (n = 30).

5) SCI + co-ultraPEALut: mice were subjected to SCI and administered co-ultraPEALut at the final dose of 1 mg/kg intraperitoneally at 1 and 6 hours after SCI (n = 30).

In a separate set of experiments to investigate the motor score, additional animals (n = 10 animals/group) were divided into the same groups and sacrificed 10 days after SCI.

### Tissue processing and light microscopy

For histopathological examination by standard hematoxylin and eosin (H&E) staining, 24 hours after injury, mice were deeply anesthetized with pentobarbital sodium and then perfused transcardially with cold PBS (0.1 M). Tissues were removed under magnified vision and segments containing the lesion (1 cm on each side of the lesion) were collected in 4% paraformaldehyde for proper fixation, and then processed and embedded in paraffin wax. Sections of 5 μm thickness were cut into longitudinal sections for the posterior area of the spinal cord, stained with H&E and studied using light microscopy (Dialux 22, Leitz, Milan, Italy). Representative images were shown. Damaged neurons were counted and the histopathological changes of the gray matter were scored on a 6-point scale: 0, no lesion observed; 1, gray matter contained one to five eosinophilic neurons; 2, gray matter contained five to ten eosinophilic neurons; 3, gray matter contained more than ten eosinophilic neurons; 4, small infarction (less than one third of the gray matter area); 5, moderate infarction (one third to one half of the gray matter area); and 6, large infarction (more than half of the gray matter area). The scores from all the sections from each spinal cord were averaged to give a final score for the individual mice.

### Grading of motor disturbance

Motor function was evaluated 10 days after SCI by open-field testing using the methodology of the Basso Mouse Scale (BMS) score on postoperative days, as described by Basso *et al*. [19].

## Materials

Unless otherwise stated, all compounds were obtained from Sigma-Aldrich. All other chemicals were of the highest commercial grade available. All stock solutions were prepared in non-pyrogenic saline (0.9% NaCl, Baxter, Milan, Italy). Tissue preparation was performed under aseptic conditions using sterile instruments.

### Statistical evaluation

All values in the figures and text are expressed as mean ± standard error of the mean (SEM) of n observations. For the *in vivo* studies, n represents the number of animals studied. In the experiments involving histology or immunohistochemistry, the figures shown are representative of at least three experiments performed on different experimental days. The results were analyzed by one-way analysis of variance (ANOVA) followed by a Bonferroni post-hoc test for multiple comparisons. BMS data were analyzed by the Mann–Whitney test. A *P* value of less than 0.05 was considered significant.

## Results

### Cell viability in spinal cord slices

**Figure 2:**
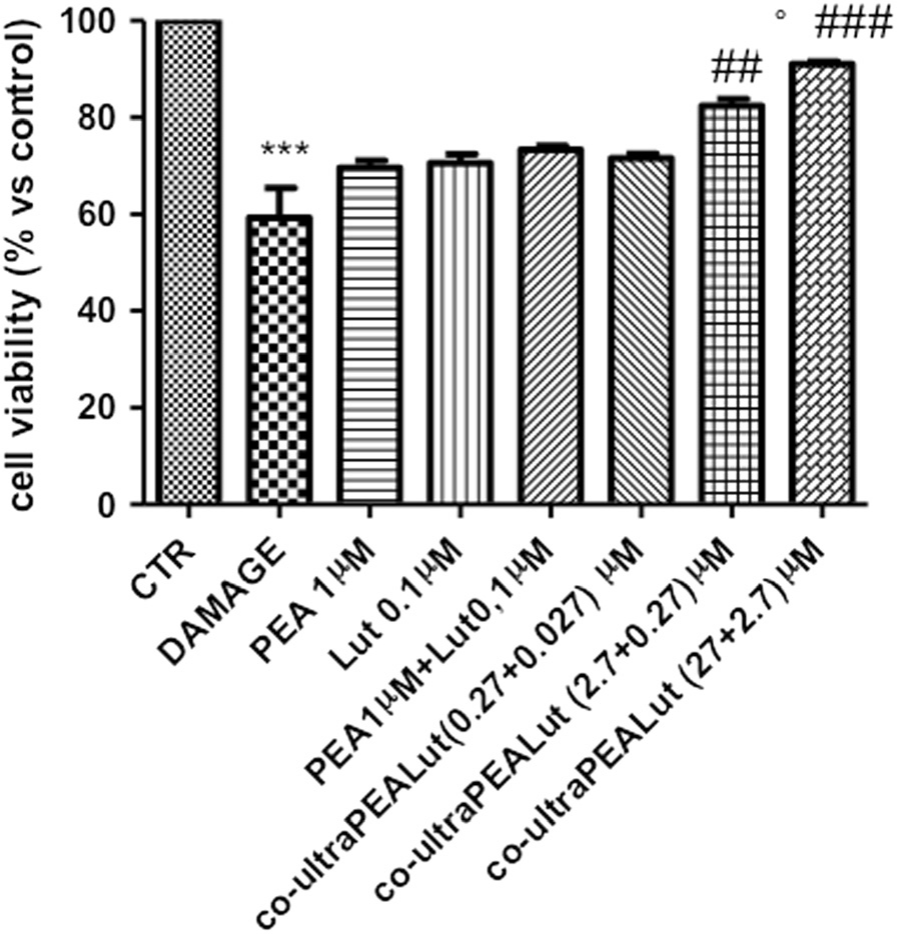
**Percentage of cell death in spinal cord injury (SCI) slices.** Viable cells within the slices, identified using MTT tetrazolium dye, were visualized under light microscopy. The level of cell death was assessed in each slice at 7 days. Cell death in injured groups was significantly higher (40%) in comparison to the control group. Pretreatment with PEA and Lut alone or with the PEA + Lut association (given as combination therapy) were not able to reduce cell death. Indeed, cell death was significantly attenuated by co-ultraPEALut pretreatment at 0.0009 g/l (containing PEA and Lut at 2.7 and 0.27 μM, respectively) and 0.009 g/l (containing PEA and Lut at 27 and 2.7 μM, respectively). This figure is representative of at least three experiments performed on different experimental days. ***P <0.001 versus Ctr; ^##^*P* <0.01 and ^###^*P* <0.001 versus Damage; °*P* <0.01 versus PEA 1 μM + Lut 0.1 μM. Ctr, control; Lut, luteolin; MTT, 3-(4,5- dimethylthiazol-2-yl)-2,5-diphenyltetrazolium bromide; PEA, palmitoylethanolamide; SCI, spinal cord injury.

Slices were successfully cultured for up to 7 days. During this time, slices preserved their morphological and structural integrity with clear differentiation of white and gray matter (data not shown). Numerous cells with multiple processes and with typical astrocyte morphology were also present within the white matter. Viable cells within the slices, identified using MTT tetrazolium dye, were visualized under light microscopy. The level of cell death was assessed in each slice at 24 hours after damage. Spinal cord organotypic slice cultures were treated with PEA (1 μM) and Lut (0.1 μM) given individually. Spinal cord slice cultures were also treated with the association of PEA + Lut (as single treatment combination) at the concentration of 1 and 0.1 μM, respectively (concentration to maintain the ratio 10:1 of PEA and Lut). Moreover, the slices were treated with a composite co-ultraPEALut at 0.00009, 0.0009 and 0.009 g/l for 24 hours. Mechanical damage induced a significant reduction in viability compared to the uninjured group (about 40% cell death). Pretreatment with PEA (1 μM) and Lut (0.1 μM) applied alone were not able to reduce cell death (Figure 2). However, the pretreatment with co-ultraPEALut (2.7 + 0.27 μM) and co-ultraPEALut (27 + 2.7 μM) significantly reduced cell death compared to the damage group (Figure 2). Moreover, we clearly showed that the association of PEA + Lut, given separately but at the same ratio of 10:1 of the co-ultraPEALut composite, was not able to counteract the damage as well as the co-ultraPEALut (Figure 2). Co-ultraPEALut (0.27 + 0.027 μM) showed a trend of reduction (Figure 2). We clearly showed that the co-ultraPEALut had greater efficacy to reduce cell death than PEA and Lut treatment given alone or as combination therapy.

### Effect of co-ultraPEALut on cyclooxygenase-2 (COX-2) expression

**Figure 3:**
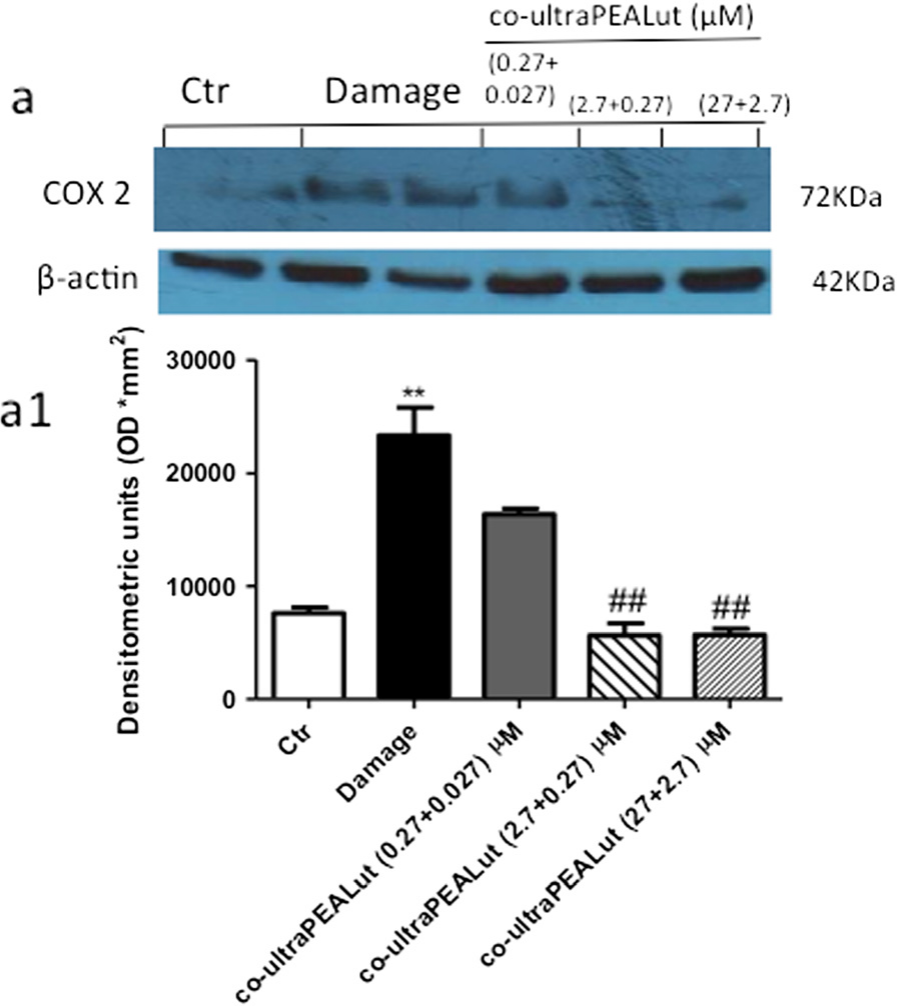
**Effect of co**-**ultraPEALut on COX**-**2 expression.** Homogenized spinal cord organotypic cultures were evaluated by western blot analysis, collected 24 hours after injury. **(a**, **a1)** Injury caused a significant increase in COX-2 expression compared to the control group. The pretreatment with co-ultraPEALut at 0.0009 g/l (containing PEA and Lut at 2.7 and 0.27 μM, respectively) and 0.009 g/l (containing PEA and Lut at 27 and 2.7 μM, respectively) significantly reduced COX-2 levels. Data were normalized on the basis of β-actin levels. This figure is representative of at least three experiments performed on different experimental days. **P <0.01 versus Ctr; ^##^*P* <0.01 versus Damage. Ctr, control; COX-2, cyclooxygenase-2; PEA, palmitoylethanolamide; Lut, luteolin.

To study the involvement of the inflammatory process following injury, we examined the ability of co-ultraPEALut to influence injury-induced COX-2 expression. Western blot analysis showed that mechanical damage significantly increased the expression of COX-2; however, the expression was markedly attenuated in spinal cord organotypic cultures pretreated with co-ultraPEALut (2.7 + 0.27 μM) and co-ultraPEALut (27 + 2.7 μM) (Figure 3). Indeed, PEA (1 μM) and Lut (0.1 μM) alone were not able to reduce COX-2 expression (data not shown).

### Effect of co-ultraPEALut on iNOS, nNOS and nitrite (NO_2_^-^) concentration

**Figure 4:**
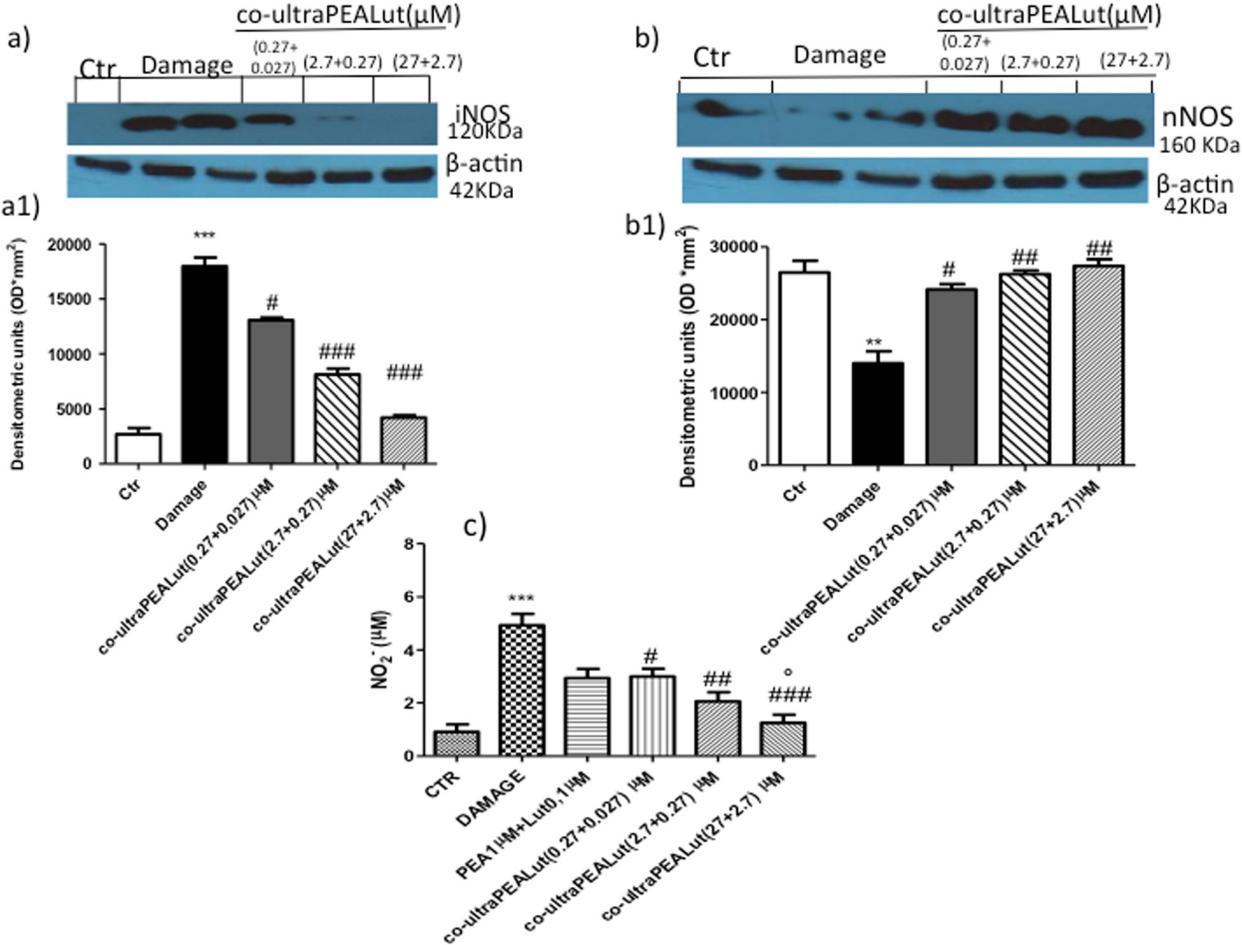
**Effect of co**-**ultraPEALut treatment on iNOS**, **nNOS and nitrite concentration.** iNOS expression was evaluated by western blot analysis in the spinal cord organotypic cultures, collected 24 hours after injury. **(a**, **a1)** iNOS expression was significantly elevated in the injured group compared to the control group. On the contrary, iNOS expression was also significantly reduced by pretreatment of co-ultraPEALut at the three different concentrations. This figure is representative of at least three experiments performed on different experimental days. ^***^P <0.001 versus Ctr; ^#^*P* <0.05 and ^###^*P* <0.001 versus Damage. We also evaluated nNOS expression by western blot analysis, which was significantly reduced by damage. **(b**, **b1)** Co-ultraPEALut treatments significantly restored the expression of nNOS. ^**^P <0.01 versus Ctr; ^#^*P* <0.05 and ^##^*P*< 0.01 versus Damage. Moreover, we evaluated the nitrite formation by nitrite assay on medium. **(c)** An increased formation of nitrite levels was evident in the injured group, while the pretreatment with co-ultraPEALut decreased the injury-induced NO production; indeed, the pretreatment with PEA + Lut association (given as combination therapy) was not able to reduce the increased NO formation as well as the treatment with PEA and with Lut administered alone (data not shown). ^***^P <0.001 versus Ctr; ^#^*P* < 0.05, ^##^*P* <0.01 and ^###^*P* <0.001 versus Damage; °*P* <0.01 versus PEA 1 μM + Lut 0.1 μM. Ctr, control; iNOS, inducible nitric oxide synthase; Lut, luteolin; nNOS, neuronal nitric oxide synthase; NO, nitric oxide; PEA, palmitoylethanolamide.

Western blot analysis also showed a significant increase in the expression of iNOS 24 hours after damage; however, pretreatment with co-ultraPEALut (0.00009, 0.0009 and 0.009 g/l) significantly attenuated the expression in a concentration-dependent manner (Figure 4a,a1). We also examined the expression of nNOS in spinal cord organotypic culture homogenates by western blot analysis. Co-ultraPEALut pretreatment at all three concentrations used in this study restored the expression of nNOS (Figure 4b,b1). Moreover, we investigated the levels of nitrite released into the culture medium by Griess reagent. The untreated control group released low levels of NO_2_^-^; instead, damage significantly enhanced the levels of NO_2_^-^ production (Figure 4c). Co-ultraPEALut treatment decreased the injury-induced NO production in the medium in a concentration-dependent manner (Figure 4c). However, the PEA + Lut association, given separately but at the same ratio of 10:1, was not able to counteract the NO production compared to the co-ultraPEALut (Figure 4c); PEA (1 μM) and Lut (0.1 μM) alone were not able to modify nNOS expression and NO production (data not shown).

### Effects of co-ultraPEALut treatment on PPAR expression

**Figure 5:**
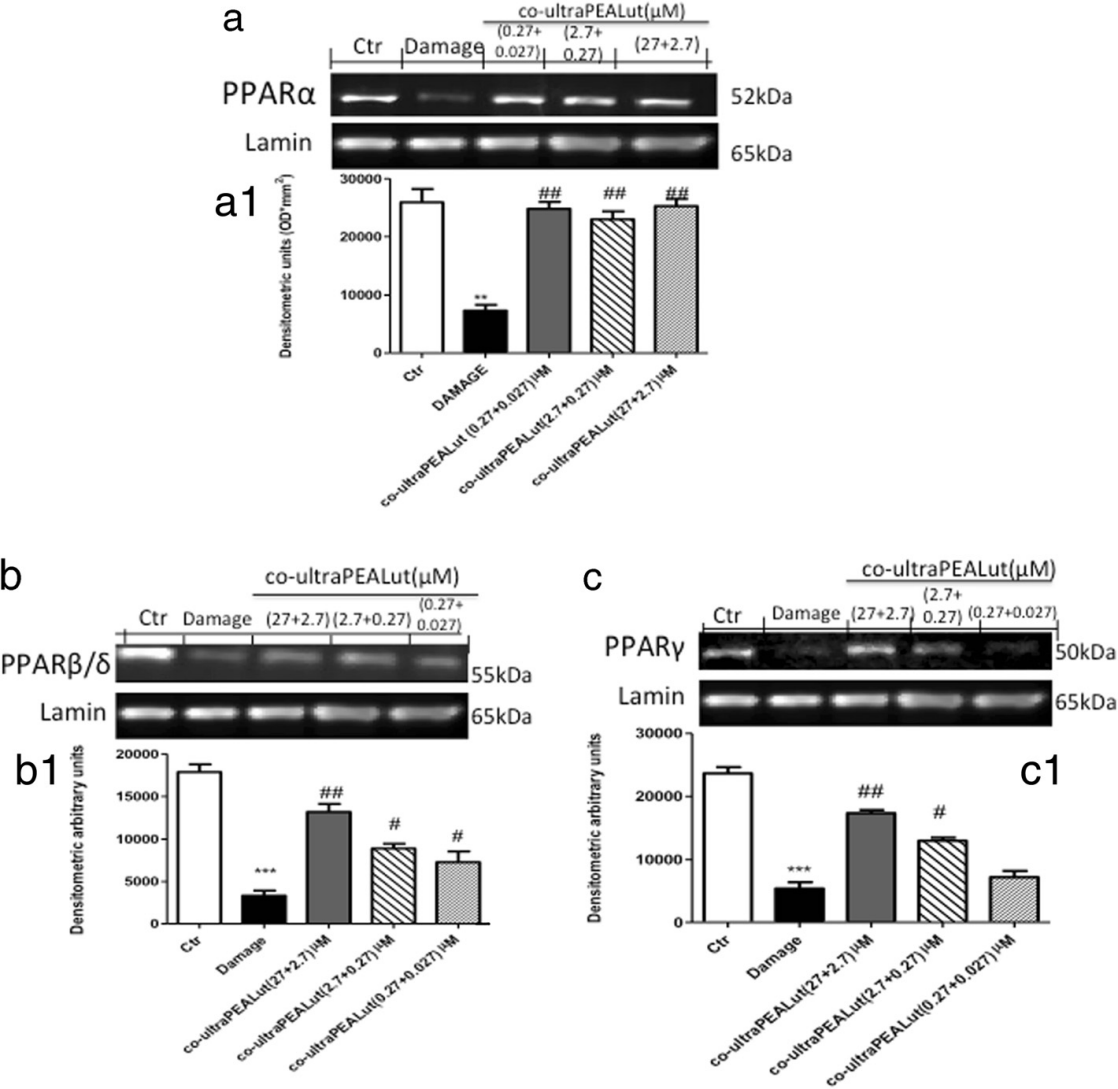
**Effect of co-ultraPEALut on PPARα, PPARβ/δ and PPARγ expression.** PPARα, PPARβ/δ and PPARγ expression were evaluated by western blot analysis. The mechanical damage induced a decrease in **(a**,**a1)** PPARα , **(b**,**b1)** PPARβ/δ and **(c**,**c1)** PPARγ expression compared to the control group. Co-ultraPEALut pretreatment at the three concentrations significantly restored the levels of both PPARα and PPARβ/δ; PPARγ expression was restored by co-ultraPEALut 0.0009 g/l (containing PEA and Lut at 2.7 and 0.27 μM, respectively) and 0.009 g/l (containing PEA and Lut at 27 and 2.7 μM, respectively). **P <0.01 and ***P <0.001 versus Ctr; ^#^*P* <0.05 and ^##^*P* <0.01 versus Damage. Ctr, control; Lut, luteolin; PEA, palmitoylethanolamide; PPAR, peroxisome proliferator-activated receptor.

To assess the mechanism of action of co-ultraPEALut, we analyzed the expression of all three isoforms of PPARs, PPARα, PPARβ/δ and PPARγ, by western blot analysis. PPARα, PPARβ/δ and PPARγ were constitutively expressed, and mechanical damage induced a significant reduction in the expression of all three isoforms (Figure 5). The pretreatment with co-ultraPEALut (2.7 + 0.27 μM) and co-ultraPEALut (27 + 2.7 μM) resulted in a significantly increased expression of PPARα, PPARβ/δ and PPARγ. Co-ultraPEALut (0.27 + 0.027 μM) significantly restored the expression of PPARα and PPARβ/δ (Figure 5a,a1), while it had no effect on PPARγ (Figure 5c,c1).

PEA (1 μM) and Lut (0.1 μM) alone had no effect on damage-induced PPAR down-regulation (data not shown).

### Co-ultraPEALut treatment reduces the severity of spinal cord trauma

**Figure 6:**
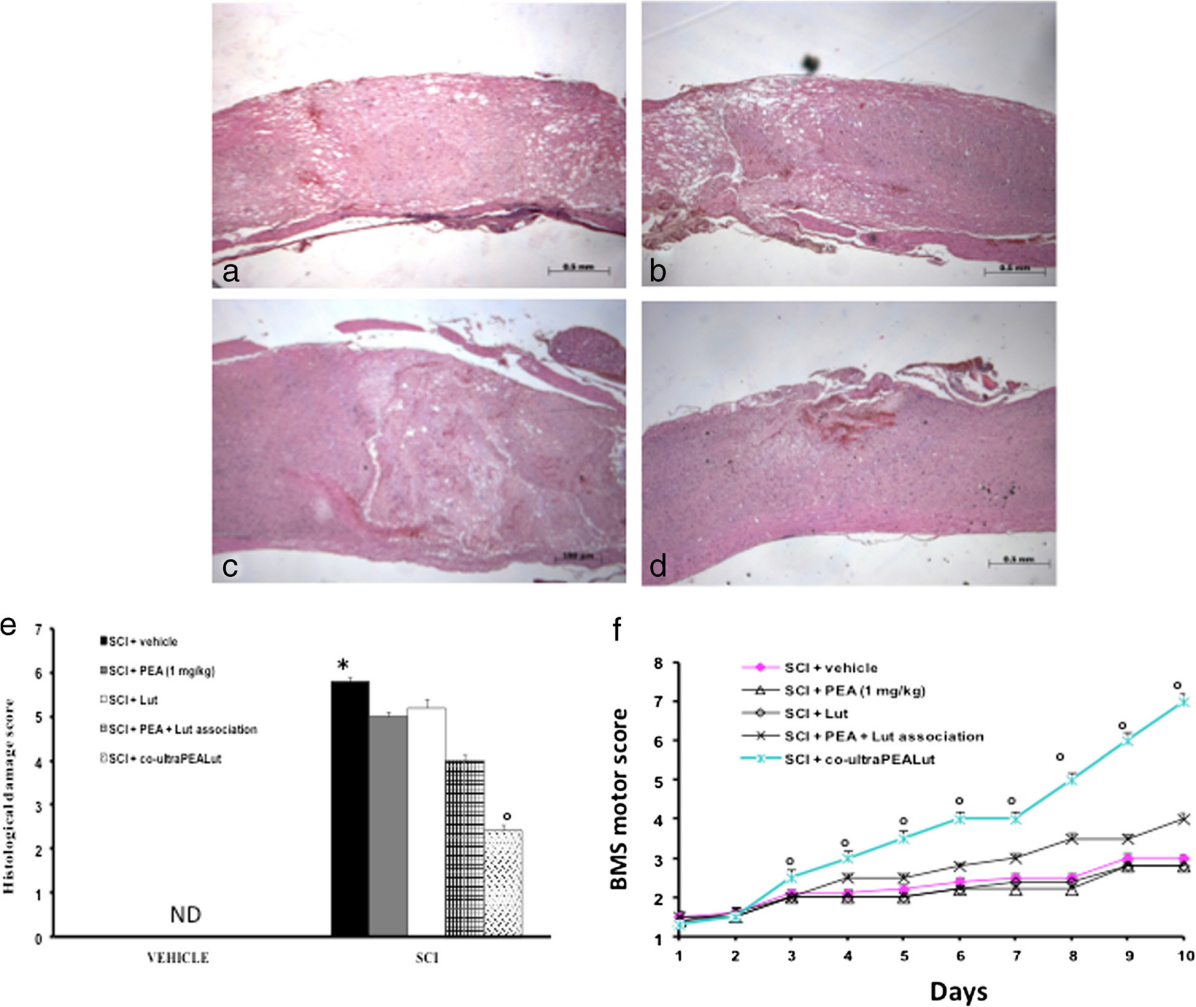
**Effect of co-ultraPEALut treatment on histological alterations of the spinal cord tissue 24 hours after injury.** Spinal cord samples were collected from the perilesional area 24 hours after injury and stained with H&E. Significant damage to the spinal cord was assessed in SCI-operated mice stained with H&E **(a**, histological score **e)** compared with sham-operated mice (data not shown). Low protection from SCI was observed in the tissue collected from mice treated with PEA at the dose of 1 mg/kg **(b**, histological score **e)**, while a strong and important protection on the severity of trauma was observed following treatment with the co-ultramicronized composite co-ultraPEALut at the dose of 1 mg/kg **(d**, histological score **e)**. Indeed, the treatment of PEA + Lut association (administered as combination therapy) was not able to protect the tissue damage induced by SCI **(c**, histological score **e)**. The histological score was made by an independent observer. Values expressed as mean ± SEM of ten mice for each group. *P <0.001 versus sham; °*P* <0.01 versus SCI. Moreover, the degree of motor disturbance was assessed every day until 10 days after SCI by Basso Mouse Scale (BMS) open-field score. Treatment with the co-ultraPEALut at the dose of 1 mg/kg reduced the motor disturbance after SCI more effectively than the treatment with PEA and Lut administered alone **(f)**, or with the pretreatment of the association of PEA + Lut administered as combination therapy, **(f)**. This figure is representative of at least three experiments performed on different experimental days. Values expressed as mean ± SEM of ten mice for each group. *P <0.01 versus sham; °*P* <0.01 versus SCI. BMS, Basso Mouse Scale; H&E, hematoxylin and eosin; Lut, luteolin; ND, not detectable; SCI, spinal cord injury; SEM, standard error of the mean; PEA, palmitoylethanolamide.

The severity of the trauma at the level of the perilesional area, assessed by the presence of edema as well as alteration of the white matter and infiltration of leukocytes, was evaluated 24 hours after injury and stained with H&E. Significant damage was observed in the spinal cord tissue collected from SCI (Figure 6a, histological score e) compared with sham-operated mice (data not shown). Indeed, a significant and important decrease in the severity of trauma was observed in mice treated with co-ultraPEALut (Figure 6d, histological score e). However, treatment with PEA (1 mg/kg) alone, or with Lut administered alone, did not modify histological damage (data not shown) as well as the PEA + Lut association, given as single treatment combination (Figure 6b and c, respectively, histological score e).

In order to evaluate histological damage to the spinal cord associated with a loss of motor function, the BMS hind limb locomotor rating score was evaluated. Mice subject to SCI had significant deficits in movement (Figure 6f). Treatment of animals with PEA alone or with Lut alone were not able to ameliorate the functional deficits induced by SCI (Figure 6f), and the PEA + Lut association, given as single treatment combination, did not restore the motor function. Indeed, co-ultraPEALut-treated mice displayed an improvement in motor activity compared to the injured group and to the other treatments (Figure 6f).

## Discussion

Damage occurring to the spinal cord following traumatic injury is due to secondary effects of glutamate excitotoxicity, pro-inflammatory cytokine expression and oxidative stress; three mechanisms that take part in a spiraling interactive cascade, ending in neuronal dysfunction and death [20]. The inflammation process, a clear consequence of the mechanical and traumatic damage that occurs after SCI, is followed by a significant production of free radicals, such as hydroxyl radicals (OH^-^ and H_2_O_2_) and NO [21,22].

In a previous study, we showed that PEA treatment (10 mg/kg) exerted beneficial effects in a model of mouse spinal cord compression, demonstrating anti-inflammatory and neuroprotective properties of PEA. Numerous data in literature reported that PEA has potent anti-inflammatory and neuroprotective effects [23,24], while lacking any direct antioxidant activity. Based on this evidence, we decided to use an association of PEA with flavonoids, such as luteolin; thus, we tested a new composite, consisting of the co-ultramicronized composite ‘co-ultraPEALut’, in a model of spinal cord organotypic cultures and an *in vivo* model.

In this study, using spinal cord organotypic slice cultures, we showed a significant decrease in cell death following co-ultraPEALut pretreatment. Cell death and neuron degeneration, which occur after the injury, are a consequence of an increase in reactive oxygen species (ROS) and NO production. NO is a unique molecule involved in a variety of physiological processes in the CNS [25]. The effects of NO on the spinal cord depend on several factors, such as concentration of NO production, activity of different synthase isoforms, cellular source of production and time of release [26]. It has been shown that low concentrations may play a role in physiological processes, while large amounts of NO may be detrimental by increasing oxidative stress. Thus, in this study we analyzed the production of NO and the expression of the different synthase isoforms, such as iNOS and nNOS by western blot analysis. We clearly demonstrated an increased iNOS expression after the injury, while the pretreatment with co-ultraPEALut was able to reduce the increase in this pro-inflammatory enzyme. Moreover, we also analyzed the expression of constitutive nNOS. Mechanical damage resulted in a loss in nNOS expression after the injury and the pretreatment with co-ultraPEALut restored the expression of this constitutive isoform.

To gain a clearer understanding of the compound’s mechanism of action, we also analyzed the expression of PPARs, based on the knowledge of the constitutive presence of PPARs on spinal cord tissue and our previous study, in which we demonstrated that PEA modified all three subtypes [23]. Thus, in this study we analyzed the nuclear expression of PPARs by western blot analysis and clearly demonstrated that the pretreatment with co-ultraPEALut restored the basal expressions.

We also tested the protective effects of the co-ultraPEALut in an *in vivo* model of spinal cord compression injury in mice. This was induced by an extradural compression of the spinal cord (T6 to T7) using an aneurysm clip to replicate the persistence of cord compression commonly observed in human SCI. After damage, animals were treated with co-ultraPEALut at a dose of 1 mg/kg. Inflammatory responses are a major component of secondary injury, and play a central role in regulating the pathogenesis of acute and chronic SCI [27]. It has been reported that reducing inflammation decreases secondary degeneration and the functional deficit after SCI. SCI resulted in tissue edema and loss of myelin in lateral and dorsal funiculus, and this histological damage was associated with the loss of motor function. At 24 hours after injury, we analyzed the severity of the trauma at the level of the perilesional area by H&E staining. Our results clearly demonstrated important damage in the spinal cord tissue collected from SCI animals compared with sham-operated mice. Protection against tissue damage and edema formation was observed in the group of mice treated with co-ultraPEALut.

Moreover, motor disturbance was assessed every day until 10 days after SCI using the BMS score. Treatment with co-ultraPEALut reduced the degree of motor disturbance more effectively than PEA treatment alone.

In conclusion, the search for molecules that participate in the neuroprotection and local restorative processes has become important, particularly in view of the potential implication to protect nervous tissue from secondary neurodegenerative events and triggering neurodegeneration. In this study, we have shown that a new composite consisting of co-ultramicronized PEA and Lut (co-ultraPEALut) exerts a protective role in response to inflammation-associated SCI. Of note, a dietary food for special medical purposes by Epitech Group, Saccolongo, Italy, whose active ingredient is a co-ultramicronized PEALut, has recently become available in some European countries for neuroinflammatory conditions.

## Conclusions

These data show new and important neuroprotective effects of the co-ultraPEALut compound, due to a combination of anti-inflammatory properties of PEA and the antioxidant capacity of Lut. These findings suggest that this composite may provide an effective strategy to treat neuroinflammation associated to SCI.

## Abbreviations

ANOVA: Analysis of variance; BMS: Basso mouse scale; CNS: Central nervous system; COX-2: Cyclooxygenase-2; Ctr: Control; DMSO: Dimethyl sulfoxide; DSC: Differential scanning calorimeter; ECL: Enhanced chemiluminescence; EDTA: Ethylenediaminetetraacetic acid; EEC: European Economic Community; ELISA: Enzyme-linked immunosorbent assay; H&E: Hematoxylin and eosin; HEPES: 4-(2-Hydroxyethyl)-1-piperazineethanesulfonic acid; IgG: Immunoglobulin G; iNOS: Inducible nitric oxide synthase; ip: Intraperitoneally; Lut: Luteolin; MEM: Modified Eagle’s medium; MTT: 3-(4,5- Dimethylthiazol-2-yl)-2,5-diphenyltetrazolium bromide; NF: Nuclear factor; nNOS: Neuronal nitric oxide synthase; NO: Nitric oxide; OD550: Optical density at 550 nm; PBS: Phosphate-buffered saline; PEA: Palmitoylethanolamide; PMT: PBS-Milk-Tween; PPAR: Peroxisome proliferator-activated receptor; ROS: Reactive oxygen species; SCI: Spinal cord injury; SEM: Standard error of the mean; TBI: Traumatic brain injury; XRD: X-ray diffraction.

## Competing interests

The authors declare that they have no competing interests.

## Authors’ contributions

IP drafted the manuscript and undertook western blot analysis; RD performed immunohistochemical analysis; DI carried out the *in vivo* experiments; MN performed statistical analysis; SC conceived of the study, and participated in its design and coordination; and EE conceived of the study, participated in its design and undertook western blot analysis. All authors read and approved the final manuscript.
