# Erratum to: A new co-ultramicronized composite including palmitoylethanolamide and luteolin to prevent neuroinflammation in spinal cord injury

**DOI:** 10.1186/s12974-016-0593-8

**Published:** 2016-05-31

**Authors:** Irene Paterniti, Daniela Impellizzeri, Rosanna Di Paola, Michele Navarra, Salvatore Cuzzocrea, Emanuela Esposito

**Affiliations:** Department of Biological and Environmental Sciences, University of Messina, Viale Ferdinando Stagno D’Alcontres, 98166 Messina, Italy; Pharmaco-Biological Department, University of Messina, Viale Annunziata, 98100 Messina, Italy

## Erratum

The authors would like to issue an erratum for this article [1] and declare the following competing interests, which we inadvertently failed to include in our original publication. The authors would like to apologise for this omission.

## Notes

The present study provides an important advance over an earlier report in which almitoylethanolamide alone was used [2], in that the co-ultramicronized almitoylethanolamide/luteolin composite demonstrates improved potency by an approximate order of magnitude.
